# Dihydroergotamine inhibits the vasodepressor sensory CGRPergic outflow by prejunctional activation of α_2_-adrenoceptors and 5-HT_1_ receptors

**DOI:** 10.1186/s10194-018-0869-8

**Published:** 2018-05-25

**Authors:** Abimael González-Hernández, Jair Lozano-Cuenca, Bruno A. Marichal-Cancino, Antoinette MaassenVanDenBrink, Carlos M. Villalón

**Affiliations:** 1 Departamento de Farmacobiología, Cinvestav-Coapa, Tenorios 235, Col. Granjas-Coapa, Deleg. Tlalpan, 14330 Ciudad de México, México; 2 Departamento de Neurobiología del Desarrollo y Neurofisiología, Instituto de Neurobiología, Universidad Nacional Autónoma de México, Campus UNAM, Juriquilla, México; 3 Departamento de Fisiología y Farmacología, Centro de Ciencias Básicas, Universidad Autónoma de Aguascalientes, Ciudad Universitaria, 20131 Aguascalientes, Ags México; 4 Division of Vascular Medicine and Pharmacology, Erasmus University Medical Center, P.O. Box 2040, 3000 CA Rotterdam, The Netherlands

**Keywords:** CGRP, Dihydroergotamine, Pithed rat, Sensory neurons, Vasodepressor responses

## Abstract

**Background:**

Dihydroergotamine (DHE) is an antimigraine drug that produces cranial vasoconstriction and inhibits trigeminal CGRP release; furthermore, it inhibits the vasodepressor sensory CGRPergic outflow, but the receptors involved remain unknown. Prejunctional activation of α_2A/2C_-adrenergic, serotonin 5-HT_1B/1F_, or dopamine D_2_-like receptors results in inhibition of this CGRPergic outflow. Since DHE displays affinity for these receptors, this study investigated the pharmacological profile of DHE-induced inhibition of the vasodepressor sensory CGRPergic outflow.

**Methods:**

Pithed rats were pretreated i.v. with hexamethonium (2 mg/kg·min) followed by continuous infusions of methoxamine (20 μg/kg·min) and DHE (3.1 μg/kg·min). Then, stimulus-response curves (spinal electrical stimulation; T_9_-T_12_) or dose-response curves (i.v. injections of α-CGRP) resulted in frequency-dependent or dose-dependent decreases in diastolic blood pressure.

**Results:**

DHE inhibited the vasodepressor responses to electrical stimulation (0.56–5.6 Hz), without affecting those to i.v. α-CGRP (0.1–1 μg/kg). This inhibition by DHE (not produced by the methoxamine infusions): (i) was abolished by pretreatment with the combination of the antagonists rauwolscine (α_2_-adrenoceptor; 310 μg/kg) plus GR127935 (5-HT_1B/1D_; 31 μg/kg); and (ii) remained unaffected after rauwolscine (310 μg/kg), GR127935 (31 μg/kg) or haloperidol (D_2_-like; 310 μg/kg) given alone, or after the combination of rauwolscine plus haloperidol or GR127935 plus haloperidol at the aforementioned doses.

**Conclusion:**

DHE-induced inhibition of the vasodepressor sensory CGRPergic outflow is mainly mediated by prejunctional rauwolscine-sensitive α_2_-adrenoceptors and GR127935-sensitive 5-HT_1B/1D_ receptors, which correlate with α_2A/2C_-adrenoceptors and 5-HT_1B_ receptors, respectively. These findings suggest that DHE-induced inhibition of the perivascular sensory CGRPergic outflow may facilitate DHE’s vasoconstrictor properties resulting in an increased vascular resistance.

**Electronic supplementary material:**

The online version of this article (10.1186/s10194-018-0869-8) contains supplementary material, which is available to authorized users.

## Background

Dihydroergotamine (DHE) is a primary drug effective in the acute treatment of migraine [1–3] and its therapeutic effect may involve: (i) cranial vasoconstriction via vascular 5-HT_1B_ and α_2A/2C_-adrenoceptors [4]; (ii) inhibition of neurogenic cranial vasodilatation produced by trigeminal release of calcitonin gene-related peptide (CGRP) [5, 6]; and probably (iii) inhibition of trigeminal nociceptive reflexes [7, 8]. More recently, DHE has been shown to increase diastolic blood pressure (an *index* of peripheral vascular resistance) by activation of vascular α_1_ (α_1A_, α_1B_ and α_1D_) and α_2_ (α_2A_, α_2B_ and α_2C_)-adrenoceptors [9]. Interestingly, at peripheral level, CGRP released from primary sensory perivascular nerves induces vasodepressor responses [10–12], but this neuropeptide does not seem to be involved in the physiological regulation of blood pressure [13]. Notwithstanding, evidence is now growing suggesting that CGRP has a protective role in the generation of hypertension, which is most likely mediated via its effects at peripheral receptors [14]. Thus, the potential side-effects produced by DHE on the systemic CGRPergic transmission via its prejunctional interactions on perivascular sensory CGRPergic nerves deserve special attention [15]. Indeed, DHE is capable of inhibiting the vasodepressor responses induced by spinal stimulation of the perivascular sensory CGRPergic outflow in pithed rats [16]; however, the pharmacological profile of the receptors involved in this inhibitory action remains thus far unclear, probably because DHE displays complex pharmacological properties as it has affinity for an array of receptors [1–3, 17]. In this respect, by using selective agonists and antagonists, our group has previously shown that the rat vasodepressor sensory CGRPergic outflow (an *index* of sensory perivascular CGRP release in resistance blood vessels [12]) can be inhibited by prejunctional activation of receptors coupled to G_i/o_ proteins, including: (i) α_2_ (specifically α_2A/2C_)-adrenoceptors [18]; (ii) serotonin 5-HT_1B_ [19] and 5-HT_1F_ [20] receptors; and (iii) dopamine D_2_-like receptors [21]. Since DHE displays affinity for these receptors (see Table 1), it is reasonable to hypothesize that these receptors could be involved in DHE-induced inhibition of the vasodepressor sensory CGRPergic outflow. On this basis, the present study in pithed rats was designed to investigate: (a) whether DHE is capable of inhibiting the vasodepressor responses induced by either stimulation of the perivascular sensory CGRPergic outflow or i.v. bolus injections of exogenous α-CGRP; and (b) the pharmacological profile of the receptors involved in DHE-induced inhibition of the vasodepressor sensory CGRPergic outflow by analysing the effects of pre-treatment with the antagonists rauwolscine (α_2_-adrenoceptors), GR127935 (5-HT_1B/1D_) and haloperidol (D_2_-like).

**Table 1:** Binding affinity constants (p*K*_i_) for the α_2_-adrenergic, dopamine D_2_-like or serotonin 5-HT_1_ receptor families and their respective receptor subtypes for dihydroergotamine (DHE), rauwolscine, GR127935 and haloperidol for cloned human receptors (unless otherwise stated)

	p*K*_*i*_ values										
Receptors Ligands	α_2_-			D_2_-like			5-HT_1_				
	α _2A_	α _2B_	α _2C_	D_2_	D_3_	D_4_	1Aª	1B	1D	1E	1F
DHE	8.7^a^	8.0^a^	9.0^a^	8.2^a^	8.2^a^	8.1^a^	9.3^a^	(r)7.8^a^	8.6^a^	6.2^a^	6.9^a^
Rauwolscine	8.9^b^	8.9^b^	9.3^b^	N.D.			N.D.	6.5^c^	7.8^c^	5.5^c^	N.D.
GR127935	< 6.0 ^d,^*			N.D.			7.2 ^e^	(r)8.8^f^	8.6^g^	5.4^g^	6.4^g^
Haloperidol	5.8 ^h,^*			9.4^i^	8.5^i^	8.8^i^	N.D.				

Data taken from: ^a^[33]; ^b^[34]; ^c^[35]; ^d^[36]; ^e^[37]; ^f^[38]; ^g^[39]; ^h^[40]; ^i^[41]. All data are given as p*K*_i_ values at human recombinant receptors, except when stated otherwise: rodent (r) receptors; N.D., not determined; *These p*K*_*i*_ values are referred for the respective family receptor

## Methods

### Animals

Male Wistar normotensive rats (300–350 g) were maintained at a 12/12-h light/dark cycle (with light beginning at 07:00 h) and housed in a special room at constant temperature (22 ± 2 °C) and humidity (50%), with food and water freely available in their home cages. All animal procedures, number of animals and the protocols of the present investigation were approved by our Institutional Ethics Committee on the use of animals in scientific experiments (CICUAL Cinvestav; protocol number 507–12), and followed the regulations established by the Mexican Official Norm (NOM-062-ZOO-1999), in accordance with ARRIVE (Animal Research: Reporting In Vivo Experiments) reporting guidelines for the care and use of laboratory animals.

### General methods

Experiments were carried out in a total of 90 rats. After anaesthesia with ether and cannulation of the trachea, the rats were pithed by inserting a stainless-steel rod through the orbit and foramen magnum into the vertebral foramen [22]. Then, the animals were artificially ventilated with room air using a model 7025 Ugo Basile pump (56 strokes per min; stroke volume = 20 ml/kg), as established by Kleinman and Radford [23]. After bilateral vagotomy, catheters were placed in: (i) the left and right femoral and jugular veins, for the continuous infusions of agonists (methoxamine and DHE) and i.v. administration of the antagonists, respectively; and (ii) the left carotid artery, connected to a Grass pressure transducer (P23XL), for the recording of arterial blood pressure. Heart rate was measured with a tachograph (7P4, Grass Instrument Co., Quincy, MA, USA) triggered from the blood pressure signal. Both blood pressure and heart rate were recorded simultaneously by a model 7 Grass polygraph (Grass Instrument Co., Quincy, MA, USA). At this point, the 90 rats were divided into two main sets, so that the effects produced by the continuous infusions of methoxamine and DHE under different treatments could be evaluated on the vasodepressor responses induced by: (i) electrical stimulation of the vasodepressor sensory CGRPergic outflow (set 1; *n =* 80); and (ii) i.v. bolus injections of exogenous α-CGRP (set 2; *n =* 10). The vasodepressor stimulus-response curves and dose-response curves by electrical stimulation and exogenous α-CGRP, respectively, were elicited using a sequential schedule at 5–10 min intervals (see below) and were completed in about 50 min. Each response was elicited under unaltered values of resting blood pressure. The body temperature of each pithed rat was maintained at 37 °C by a lamp and monitored with a rectal thermometer.

### Experimental protocols

After the animals (*n* = 90) had been in a stable haemodynamic condition for at least 15 min, baseline values of diastolic blood pressure (a more accurate indicator of peripheral vascular resistance, as previously established [12, 18–21]) and heart rate were determined.

### Protocol 1. Electrical stimulation of the perivascular (vasodepressor) sensory outflow

In the first set of rats (*n* = 80), the pithing rod was replaced by an electrode enamelled except for 1.5 cm length 9 cm from the tip, so that the uncovered segment was situated at T_9_-T_12_ of the spinal cord, and an indifferent electrode was placed dorsally [16, 18–22]. Before electrical stimulation, the animals received (i.v.): (i) a bolus injection of gallamine (25 mg/kg) to avoid electrically-induced muscular twitchings; (ii) ten min later, a continuous infusion of hexamethonium (2 mg/kg·min) to block the electrically-induced vasopressor responses that are produced by stimulation of the preganglionic sympathetic vasopressor outflow; and (iii) ten min later, a continuous infusion of methoxamine (20 μg/kg·min) to produce a sustained increase in diastolic blood pressure that allows us to produce the subsequent induction of vasodepressor responses, as previously described [16, 18–21]. Ten min later, this set of rats was divided into three groups.

The first group (*n* = 10) was subdivided into two subgroups (*n* = 5 each one) that received: (i) nothing (control experiment with no vehicles; see below); and (ii) an i.v. continuous infusion of DHE (3.1 μg/kg·min), a dose that has previously been shown to produce (amongst several doses) a maximal inhibition of the vasodepressor sensory CGRPergic outflow in pithed rats [16]. Twenty minutes later, diastolic blood pressure and heart rate were determined again, and then, the vasodepressor sensory CGRPergic outflow was electrically stimulated during the above treatments to elicit vasodepressor responses by applying 10-s trains of monophasic, rectangular pulses (2 msec, 50 V), at increasing frequencies of stimulation (0.56, 1, 1.8, 3.1 and 5.6 Hz). When diastolic blood pressure had returned to baseline levels, the next frequency was applied. This procedure was systematically performed until the stimulus-response curve had been completed.

The second group (*n* = 35) received an i.v. continuous infusion of 1% propylene glycol (PPG; vehicle for dissolving DHE) (0.02 ml/min). Ten min later, this group was subdivided into seven subgroups (*n* = 5 each) comprising i.v. bolus injections of, respectively: (i) saline (1 ml/kg); (ii) rauwolscine (310 μg/kg); (iii) GR127935 (31 μg/kg); (iv) haloperidol (310 μg/kg); (v) rauwolscine+GR127935 (310 and 31 μg/kg, respectively); (vi) rauwolscine+ haloperidol (310 μg/kg, each); and (vii) GR127935 + haloperidol (31 and 310 μg/kg, respectively). After 10 min, a stimulus-response curve was constructed as described above during the infusion of methoxamine to determine the effect of these antagonists per se.

The third group (*n* = 35) received an i.v. continuous infusion of DHE (3.1 μg/kg·min). Ten min later, this group was subdivided into seven subgroups (*n* = 5 each) comprising i.v. bolus injections of, respectively: (i) saline (1 ml/kg); (ii) rauwolscine (310 μg/kg); (iii) GR127935 (31 μg/kg); (iv) haloperidol (310 μg/kg); (v) rauwolscine+GR127935 (310 and 31 μg/kg, respectively); (vi) rauwolscine+haloperidol (310 μg/kg, each); and (vii) GR127935 + haloperidol (31 and 310 μg/kg, respectively). Ten minutes later, a stimulus-response curve was constructed as described above, during the infusion of DHE.

### Protocol 2. Administration of exogenous α-CGRP

The second set of rats (*n* = 10) was prepared as describe above, but the pithing rod was left throughout the experiment and the administration of both gallamine and hexamethonium was omitted, as previously described [16, 18–21]. Then, this set received and i.v. continuous infusion of methoxamine (20 μg/kg·min); after 10 min, this set was divided into two groups (*n* = 5 each) that received, respectively: (i) nothing (control group); or (ii) an i.v. continuous infusion of DHE (3.1 μg/kg·min). Twenty min later, the values of diastolic blood pressure and heart rate were determined again, and then, the vasodepressor responses elicited by i.v. bolus injections of exogenous α-CGRP (0.1, 0.18, 0.31, 0.56 and 1 μg/kg) were examined during the infusions of methoxamine and DHE.

### Other procedures applying to protocols 1 and/or 2

The doses of hexamethonium, methoxamine and DHE were continuously infused at a rate of 0.02 ml/min by a WPI model sp100i pump (World Precision Instruments Inc., Sarasota, FL, USA). The dose of DHE was selected from a previous study [16]. The intervals between the different stimulation frequencies or doses of α-CGRP applied were dependent on the duration of the resulting vasodepressor responses (5–10 min), as we waited until diastolic blood pressure had returned to baseline values.

### Drugs

Apart from the anaesthetic (diethyl ether), the compounds used in this study (obtained from the sources indicated) were: gallamine triethiodide, hexamethonium chloride, rat α-CGRP, methoxamine hydrochloride, rauwolscine hydrochloride, (Sigma Chemical Co., St Louis, MO, USA); N-[methoxy-3-(4-methyl-1-piperazinyl)phenyl]-2′-methyl-4′-(5-methyl-1,2,4-oxadiazol-3-yl)[1,1-biphenyl]-4-carboxamidehydrochloride (GR127935) (gift from GlaxoSmithKline, Stevenage, Hertfordshire, UK); and DHE tartrate (gift from Novartis Pharma A.G., Basel, Switzerland). All compounds were dissolved in saline, except: (i) DHE, which was dissolved in propylene glycol and gauged with saline to have a final solution of 1% PPG; and (ii) haloperidol, which was dissolved in some drops of 5% ascorbic acid and the resulting solution was finally diluted with saline. These vehicles had no effect on baseline diastolic blood pressure or heart rate (data not shown). Fresh solutions were prepared for each experiment. The doses of agonists refer to their respective salts, whereas those of the antagonists refer to their free base.

### Data presentation and statistical evaluation

All data in the text, tables and figures, unless stated otherwise, are presented as mean ± standard error of the mean (S.E.M). The peak changes in diastolic blood pressure by electrical stimulation or exogenous α-CGRP were expressed as percent change from baseline, as previously reported [16, 18–21]. The difference in the absolute values of diastolic blood pressure and heart rate within one subgroup of animals before and during the continuous infusions of methoxamine (20 μg/kg·min) and DHE (3.1 μg/kg·min) were evaluated with paired Student’s *t*-test. Moreover, a one-way analysis of variance was used to compare the absolute values of diastolic blood pressure and heart rate obtained during the continuous infusions of methoxamine (20 μg/kg·min) and DHE (3.1 μg/kg·min) before, immediately after and 10 min after administration of saline or the antagonists used. Finally, the vasodepressor responses induced by electrical stimulation or exogenous α-CGRP in the different subgroups of animals were compared with a two-way analysis of variance. The one- and two-way analyses of variance were followed, if applicable, by the Student-Newman-Keuls’ test. Statistical significance was accepted at *P* < 0.05. The statistical analysis was performed using the SigmaPlot software (V 12.0; Systat Software, Inc.), whereas the graphs were made with GraphPad Prism® software (V 6.01; GraphPad Software, Inc.).

## Results

### Systemic haemodynamic effects of the different treatments

**Fig. 1:**
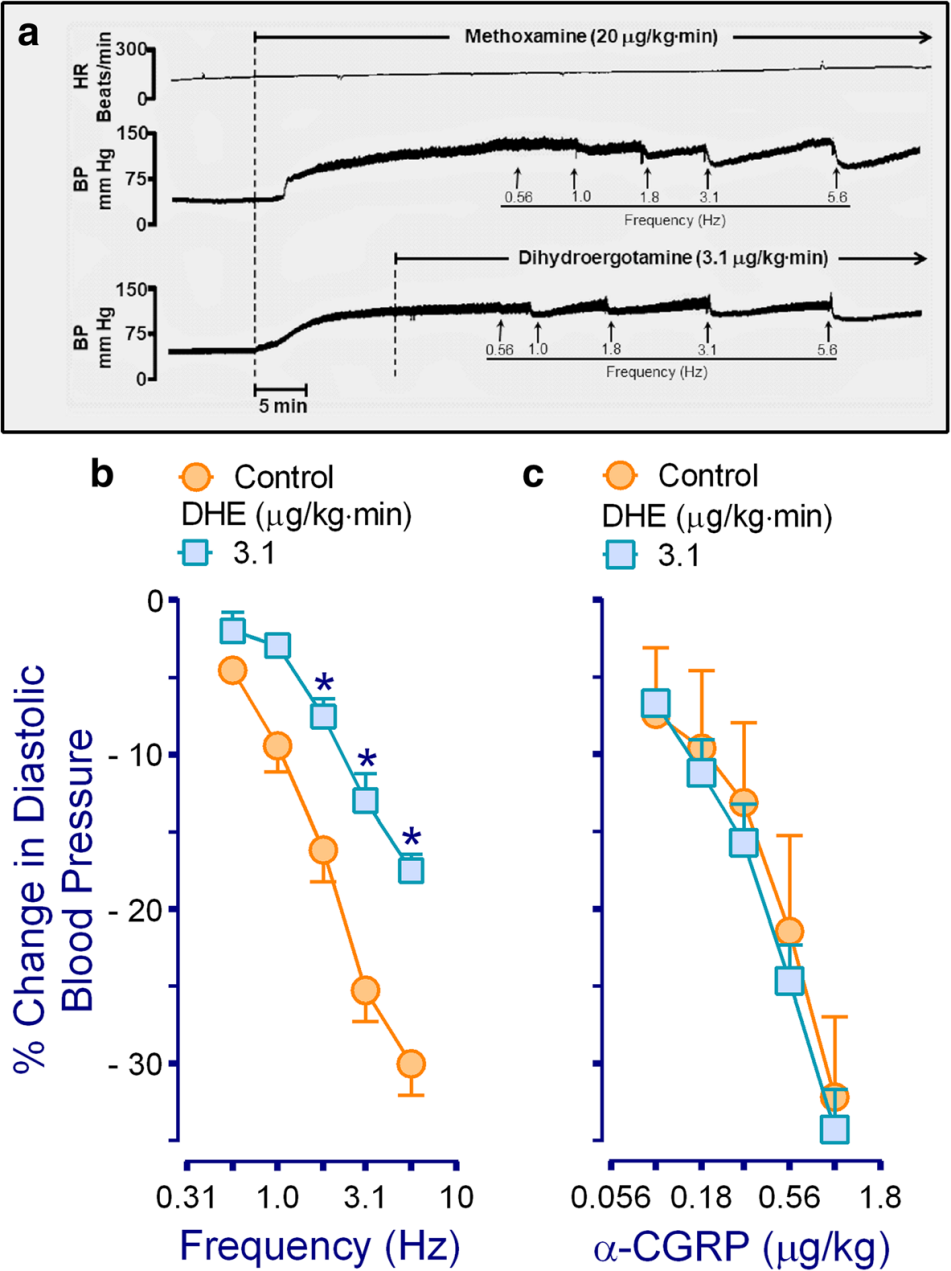
Effect of dihydroergotamine (DHE) on the vasodepressor CGRPergic outflow in pithed rats. **a** Original experimental tracings illustrating the vasodepressor responses induced by electrical stimulation of the perivascular sensory CGRPergic outflow during continuous infusions of either methoxamine (control; above) or DHE (below). Note that during continuous infusions of DHE (3.1 μg/kg·min) the vasodepressor responses induced by electrical stimulation were attenuated versus control. In both cases, the vasodepressor responses were selective as no changes in heart rate were observed. Panels (**b**) and (**c**) show the vasodepressor responses by electrical stimulation or i.v. bolus injections of α-CGRP, respectively, induced during an i.v. continuous infusions of 3.1 μg/kg·min DHE (*n* = 5 each). For the sake of clarity, control responses (○) were induced during continuous infusions of methoxamine (20 μg/kg·min). * Significantly different responses (*P* < 0.05) vs. control. BP, blood pressure; HR, heart rate

The baseline values of diastolic blood pressure and heart rate in the 90 pithed rats were 57 ± 5 mmHg and 243 ± 8 beats per min, respectively; these variables remained unchanged after gallamine or hexamethonium. Twenty min after starting the i.v. continuous infusions of methoxamine, baseline values of diastolic blood pressure and heart rate were significantly (*P* < 0.05) increased in all animals (i.e. 140 ± 4 mmHg and 273 ± 4 beats per min, respectively). It is noteworthy that during the infusions of methoxamine and/or DHE a transient, but significant, decrease in diastolic blood pressure was produced immediately after administration of an i.v. bolus injections of rauwolscine, haloperidol or the combinations of these antagonists, but not with saline or GR127935 (see Table 2). However, the values of diastolic blood pressure in the different subgroups before and 10 min after administration of saline, or antagonists, were not significantly different (*P* > 0.05) (Table 2). Furthermore, the increase in diastolic blood pressure produced by the continuous infusion of methoxamine was sustained throughout the experiments, as illustrated in Fig. 1a.

**Table 2:** Values of diastolic blood pressure and heart rate during the infusion of methoxamine (20 μg/kg·min): (i) before; (ii) immediately after (within 0–1 min after antagonist administration); and (iii) 10 min after i.v. administration of saline, rauwolscine, GR127935 and haloperidol given separately, as well as their respective combinations

Treatment	Dose (μg/kg)	n	Diastolic blood pressure (mm Hg)			Heart rate (beats per min)		
			Before	0–1 min after	10 min after	Before	0–1 min after	10 min after
Saline	1^a^	5	155 ± 9	158 ± 7	160 ± 13	260 ± 6	267 ± 3	259 ± 6
Rauwolscine (Rauw)	310	5	131 ± 5	105 ± 15*	134 ± 6	261 ± 6	257 ± 6	264 ± 5
GR127935 (GR)	31	5	137 ± 6	134 ± 7	140 ± 11	257 ± 4	251 ± 5	250 ± 5
Haloperidol (Halo)	310	5	124 ± 7	67 ± 4*	119 ± 7	234 ± 3	229 ± 3	230 ± 4
Rauw+GR	310, 31	5	168 ± 15	116 ± 13*	163 ± 11	264 ± 7	255 ± 8	271 ± 6
GR+ Halo	31, 310	5	149 ± 10	92 ± 15	119 ± 7	270 ± 10	259 ± 7	268 ± 9
Rauw+Halo	310, 310	5	123 ± 7	84 ± 6*	110 ± 3	298 ± 1	283 ± 3	300 ± 10

All values are expressed as mean ± S.E.M

^a^Saline was given at a dose of 1 ml/kg

*P < 0.05, significantly different from before. One-way analysis of variance

### Vasodepressor responses produced by electrical stimulation or exogenous α-CGRP

Figure 1a shows some representative experimental tracings illustrating that during the infusion of methoxamine the onset of the responses induced by electrical stimulation (0.56–5.6 Hz) of the vasodepressor sensory outflow (T_9_-T_12_) were immediate and resulted in frequency-dependent decreases in diastolic blood pressure. It must be emphasized that these vasodepressor responses were due to selective stimulation of the vasodepressor sensory CGRPergic outflow, since only negligible and inconsistent effects in heart rate were observed, as described earlier [16, 18–21]. In addition, as previously reported by Lozano-Cuenca et al. [16], stimulation of the vasodepressor sensory CGRPergic outflow also resulted in vasodepressor responses during the infusion of DHE (3.1 μg/kg·min), but the magnitude of these responses was clearly smaller than those elicited during the infusion of methoxamine (20 μg/kg·min).

Moreover, during the methoxamine infusion (control; 20 μg/kg·min): (i) electrical stimulation of the perivascular sensory outflow resulted in frequency-dependent vasodepressor responses, which were inhibited during the infusion 3.1 μg/kg·min DHE (see Fig. 1b); and (ii) i.v. bolus injections of exogenous α-CGRP elicited dose-dependent vasodepressor responses, but these responses, unlike those by electrical stimulation, remained unchanged during the infusion of 3.1 μg/kg·min DHE Fig. 1c). In view that 3.1 μg/kg·min DHE inhibited the electrically-induced vasodepressor responses without affecting those by exogenous α-CGRP, we considered this infusion dose of DHE for further pharmacological analysis. In all cases, the vasodepressor responses to electrical stimulation or exogenous α-CGRP: (i) appeared about 10 s after starting each electrical stimulus or dose of α-CGRP, and reached a maximum 1 min after the stimulus had ended; and (ii) returned to baseline levels within 5–10 min after each stimulus/dose, as previously reported [18].

### Effect per se of saline, rauwolscine, GR127935 or haloperidol (given separately or in combination) on the neurogenic vasodepressor responses during an infusion of methoxamine

During the methoxamine infusion (control; 20 μg/kg·min), the vasodepressor responses to electrical stimulation in control animals did not significantly differ from those elicited in animals pre-treated (see Additional file 1: Figure S1 [S1]) with an i.v. bolus injection of: (i) vehicle (1 ml/kg; Additional file 1: Figure S1a); (ii) rauwolscine (α_2_-drenoceptor antagonist, 310 μg/kg; Additional file 1: Figure S1b); (iii) GR127935 (5-HT_1B/1D_ receptor antagonist, 31 μg/kg; Additional file 1: Figure S1a); (iv) haloperidol (D_2_-like receptor antagonist, 310 μg/kg; Additional file 1: Figure S1d); (v) rauwolscine+GR127935 (310 and 31 μg/kg respectively; Additional file 1: Figure S1e); (vi) rauwolscine+ haloperidol (310 μg/kg each; Additional file 1: Figure S1f); and (vii) GR127935+ haloperidol (31 and 310 μg/kg respectively; Additional file 1: Figure S1g). These results indicate that these compounds, at the doses used and under the present experimental conditions, were essentially devoid of any effect per se on the electrically-induced vasodepressor responses.

### Effect of saline, rauwolscine, GR127935 or haloperidol (given separately or in combination) on DHE-induced inhibition of the neurogenic vasodepressor responses

**Fig. 2:**
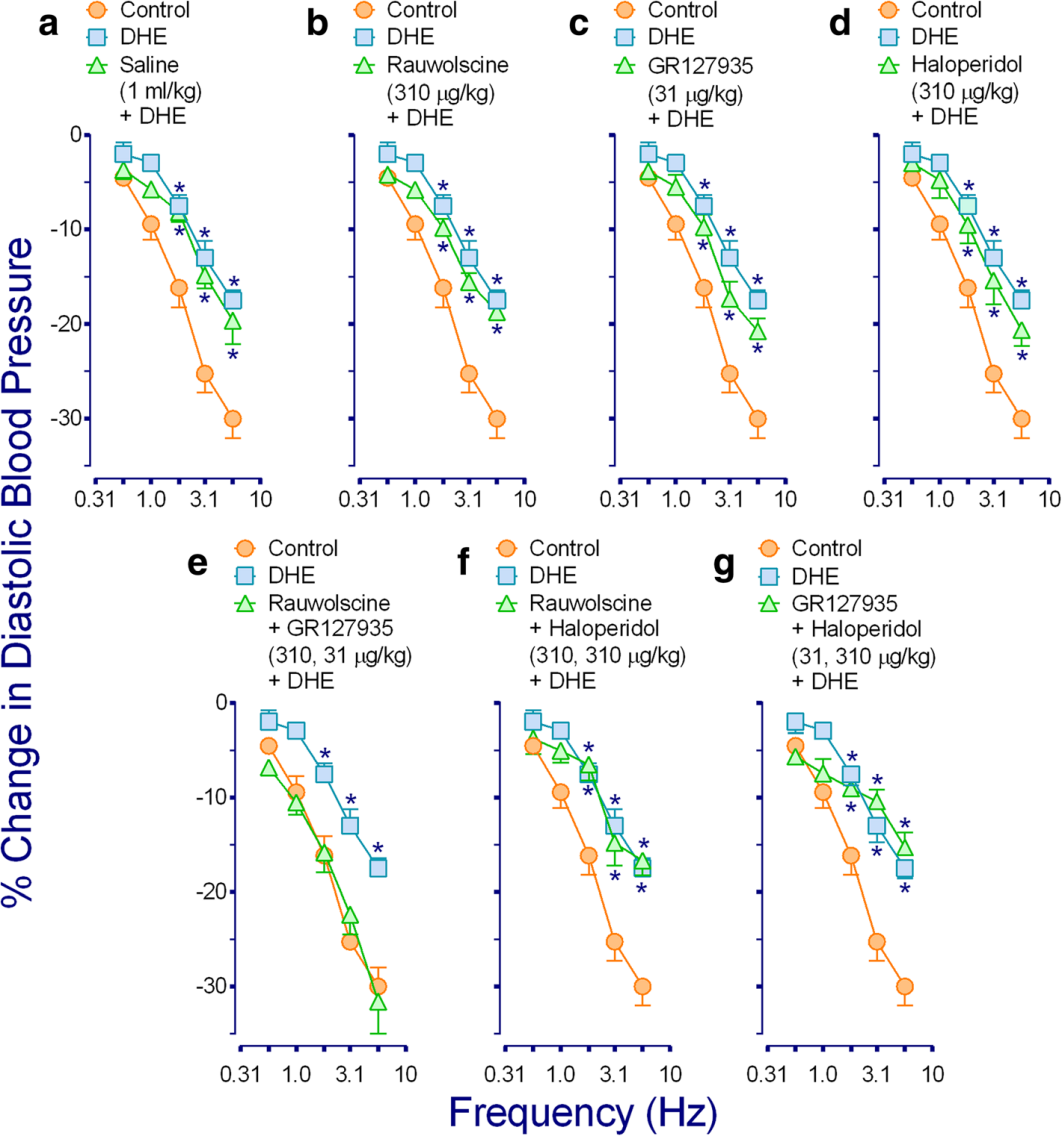
Effect of i.v. bolus injections of: (**a**) saline (1 ml/kg); (**b**) rauwolscine (310 μg/kg); (**c**) GR127935 (31 μg/kg); or (**d**) haloperidol (310 μg/kg) given separately, as well as the combinations (**e**) rauwolscine plus GR127935 (310 and 31 μg/kg, respectively); (**f**) rauwolscine plus haloperidol (310 μg/kg each); or (**g**) GR127935 plus haloperidol (31 and 310 μg/kg, respectively) on the inhibition induced by dihydroergotamine (DHE; 3.1 μg/kg·min; □) of the electrically-induced vasodepressor responses. The control responses (○) represent that of animals receiving an i.v. continuous infusion of methoxamine (20 μg/kg·min) which is shown for comparison. * Significantly different responses (*P* < 0.05) vs. control

Figure 2 shows that the inhibition induced by DHE (3.1 μg/kg·min) of the electrically-induced vasodepressor responses, which remained unaltered in animals pretreated with vehicle (1 ml/kg; Fig. 2a), was: (i) abolished in animals pretreated with rauwolscine+GR127935 (310 and 31 μg/kg respectively; Fig. 2e); and (ii) resistant to blockade in animals pretreated with rauwolscine (310 μg/kg; Fig. 2b); GR127935 (31 μg/kg; Fig. 2c); haloperidol (310 μg/kg; Fig. 2d); rauwolscine+haloperidol (310 and 310 μg/kg each; Fig. 2f); or GR127935+ haloperidol (31 and 310 μg/kg respectively; Fig. 2g).

## Discussion

### General

Apart from the implications discussed below, our study confirms that DHE can inhibit the vasodepressor sensory CGRPergic outflow in pithed rats by prejunctional mechanisms, as previously reported by Lozano-Cuenca et al. [16]. However, these authors made no attempt to identify the pharmacological profile of receptors involved in such inhibition by DHE. Hence, by using antagonists for α_2_-adrenoceptors (rauwolscine), 5-HT_1B/1D_ receptors (GR127935) and D_2_-like receptors (haloperidol) (since DHE displays affinity for these receptors; see Table 1), the present study suggests that α_2_-adrenoceptors and 5-HT_1B/1D_ receptors (but *not* D_2_-like receptors) are involved in the prejunctional mechanisms by which DHE inhibits the vasodepressor sensory CGRPergic outflow in pithed rats.

Moreover, it is important to note that we did not measure sensory nerve activity directly, but the electrically-induced CGRP release in the systemic vasculature could be estimated indirectly by measurement of the evoked vasodepressor response, as previously established using the CGRP receptor antagonists CGRP_8–37_ [12] and olcegepant [24]. Hence, the inhibition by DHE was considered sensory-inhibitory since this ergot inhibited the vasodepressor responses induced by spinal (T_9_-T_12_) stimulation of the vasodepressor sensory CGRPergic outflow (Fig. 1b), without affecting those by exogenous α-CGRP (Fig. 1c).

### Systemic haemodynamic effects produced by methoxamine and DHE

As previously established in pithed rats [16, 18–21], the artificial and sustained increase in diastolic blood pressure (at around 140 mmHg) by a continuous infusion of the α_1_-adrenoceptor agonist methoxamine (20 μg/kg·min; Fig. 1a) is a *conditio sine qua non* for inducing vasodepressor responses. Otherwise, the basal blood pressure in pithed rats is so low that there is no “window” for eliciting further decreases in this variable. The methoxamine-induced increase in diastolic blood pressure has been attributed to an increase in peripheral vascular resistance [25]. In addition, it is noteworthy that 3.1 μg/kg·min DHE can slightly increase diastolic blood pressure when the methoxamine infusion is not given (i.e. when basal diastolic blood pressure is too low; data not shown). Accordingly, the methoxamine-induced increase in blood pressure, which is maximal [16, 18], could most probably have masked the slight effect of DHE on this variable. In fact, the pressor effect of DHE has been extensively described in humans [26, 27], and its pressor effect in pithed rats has recently been associated with vascular activation of α_1_ (α_1A_, α_1B_ and α_1D_) and α_2_ (α_2A_, α_2B_ and α_2C_)-adrenoceptors [9].

### Effects of several antagonists per se on systemic haemodynamic variables and on the sensory-induced vasodepressor responses

To identify the mechanisms involved in the prejunctional inhibition by DHE (Fig. 1b and c), we decided to evaluate the effect of several antagonists per se (Table 1) on systemic haemodynamic conditions and on the vasodepressor responses induced by electrical stimulation. A transient, but significant, decrease in diastolic blood pressure was observed when animals received a bolus injection of rauwolscine and/or haloperidol (Table 2). In the case of haloperidol, this effect could be explained by considering that this compound exhibits high affinity for α_1_-adrenoceptors (p*K*_*i*_: 8.0; see Table 1). Thus, it is tempting to suggest that haloperidol may have an antagonistic effect on methoxamine (α_1_-adrenoceptor agonist)-induced increase in blood pressure. In contrast, we have no clear-cut explanation for the decreases in diastolic blood pressure induced by rauwolscine, which does not display affinity for α_1_-adrenoceptors. Nevertheless, in all cases, 10 min after administration of antagonists the values of diastolic blood pressure had returned to baseline values (Table 2; before and 10 min after). These results, coupled to the lack of effect of the above antagonists (alone or in combination) on the electrically-induced vasodepressor responses (see Additional file 1: Figure S1) indicates that these compounds, at the doses used, were devoid of any effects per se on the above variables. Accordingly, these data suggest that any effect of a given antagonist on DHE-induced sensory inhibition is due to a direct interaction of the antagonist with its respective receptors. It must be emphasized that: (i) our suggestion supporting and/or excluding the role of α_2_-adrenergic, 5-HT_1B/1D_ or D_2_-like receptors is based on the assumption that species differences between the binding of agonists and antagonists used do not play a major role (Table 1); and (ii) the doses of antagonists used were high enough to completely block prejunctional α_2_-adrenoceptors (rauwolscine; [18]), 5-HT_1B/1D_ receptors (GR127935; [19, 20, 28, 29]) and D_2_-like receptors (haloperidol; [21]) mediating inhibition of neurogenic cardiovascular responses in pithed rats.

### Role of α_2_-adrenergic and 5-HT_1B/1D_, but not D_2_-like, receptors in the inhibition by DHE

As previously pointed out, DHE displays affinity for α_2_-adrenergic, 5-HT_1_ and D_2_-like receptors (see Table 1). Activation of these receptors, which are coupled to G_*i/o*_ proteins, may inhibit adenylyl cyclase activity, inactivate Ca^2+^ channels and/or activate inwardly rectifying K^+^ channels [30, 31]. These are signal transduction systems usually associated with inhibition of neurotransmitter release [30, 31]. With this idea in mind and considering our results (Fig. 2), the simplest interpretation of these findings suggests that DHE-induced inhibition mainly involves the activation of prejunctional α_2_-adrenergic and 5-HT_1B/1D_ receptors, but not of D_2_-like receptors since the DHE response was: (i) only abolished by rauwolscine plus GR127935 (Fig. 2e); and (ii) resistant to blockade by rauwolscine (Fig. 2b), GR127935 (Fig. 2c), haloperidol (Fig. 2d), rauwolscine plus haloperidol (Fig. 2f) or GR127935 plus haloperidol (Fig. 2g). However, the lack of blockade by some of the above treatments deserves further considerations. For example, the fact that rauwolscine or GR127935 alone failed to block DHE-induced inhibition may reflect the fact that a maximal dose of DHE was used [16]; accordingly, DHE could be stimulating α_2_-adrenoceptors and 5-HT_1B/1D_ receptors simultaneously; thus, when blocking only one of these receptors, the inhibition produced by the unblocked receptor will overshadow the antagonism produced on the other receptor. In addition, the involvement of D_2_-like receptors seems unlikely based on the lack of effect of haloperidol, an antagonist with high affinity (p*K*_*i*_) for the D_2_-like receptors subtypes (D_2_: 9.4; D_3_: 8.5 and D_4_: 8.8; see Table 1). This suggestion gains weight when considering that DHE-induced inhibition remained unaffected after rauwolscine plus haloperidol (Fig. 2f) or GR127935 plus haloperidol (Fig. 2g).

Having established the main involvement of rauwolscine-sensitive α_2_-adrenoceptors and GR127935-sensitive 5-HT_1B/1D_ receptors in DHE-induced inhibition, we have to recognize that no attempt was made here to further identify the specific subtypes of these main receptor families. The reason for this omission is based on the fact that we have previously shown (using selective agonists and antagonists) that these receptors correlate with the pharmacological profile of, respectively: (i) α_2A/2C_ (but not α_2B_)-adrenoceptor subtypes [18]; and (ii) 5-HT_1B_ and 5-HT_1F_ (but not 5-HT_1A_ or 5-HT_1D_) receptor subtypes [19, 20]. However, the fact that DHE-induced inhibition was abolished by the combination rauwolscine (310 μg/kg) + GR127935 (31 μg/kg), where the latter dose is not enough to block the prejunctional 5-HT_1F_ receptors that inhibit the rat vasodepressor sensory CGRPergic outflow [20], makes the role of these subtypes rather unlikely. Finally, it is noteworthy that DHE also displays moderate affinity for other receptors, including the 5-ht_1E_ (p*K*_*i*_: 6.2) subtype (Table 1). However, the 5-ht_1E_ retains its lower-case appellation as it is not a functional receptor [32].

## Conclusion

The above results suggest that DHE-induced inhibition of the vasodepressor sensory CGRPergic outflow is mainly mediated by prejunctional activation of rauwolscine-sensitive α_2_-adrenoceptors and GR127935-sensitive 5-HT_1B/1D_ receptors which, most likely, correlate with α_2A/2C_-adrenoceptors [18] and 5-HT_1B_ receptors [19], respectively. These findings may shed further light on the vascular side effects produced by DHE, namely: DHE-induced inhibition of the perivascular sensory CGRPergic outflow may facilitate DHE’s vasoconstrictor properties resulting in an increased vascular resistance.

## Additional file

Additional file 1: **Figure S1.** Effect per se of i.v. bolus injections of: (a) saline (1 ml/kg); (b) rauwolscine (310 μg/kg); (c) GR127935 (31 μg/kg); or (d) haloperidol (310 μg/kg) given separately; as well as the combinations (e) rauwolscine plus GR127935 (310 and 31 μg/kg, respectively); (f) rauwolscine plus haloperidol (310 μg/kg each); or (g) GR127935 plus haloperidol (31 and 310 μg/kg, respectively) on the electrically-induced vasodepressor responses produced during an i.v. continuous infusion of methoxamine (20 μg/kg. min) (*n* = 5 for each group). No significant effects were produced after administration of compounds (*P* > 0.05). (PDF 881 kb)

## Abbreviations

ARRIVE: Animal Research: Reporting In Vivo Experiments
DHE: Dihydroergotamine
i.p.: Intraperitoneal
i.v.: Intravenous
PPG: Propylene glycol
α-CGRP: α-Calcitonin gene-related peptide
