# Supplementary material for: Dihydroergotamine inhibits the vasodepressor sensory CGRPergic outflow by prejunctional activation of α2-adrenoceptors and 5-HT1 receptors
Source: J Headache Pain. 2018 May 25;19(1):40. doi: 10.1186/s10194-018-0869-8 (PMC5970131; doi:10.1186/s10194-018-0869-8)
Supplement: Supplementary file 1 — Figure S1. Effect per se of i.v. bolus injections of: (a) saline (1 ml/kg); (b) rauwolscine (310 μg/kg); (c) GR127935 (31 μg/kg); or (d) haloperidol (310 μg/kg) given separately; as well as the combinations (e) rauwolscine plus GR127935 (310 and 31 μg/kg, respectively); (f) rauwolscine plus haloperidol (310 μg/kg each); or (g) GR127935 plus haloperidol (31 and 310 μg/kg, respectively) on the electrically-induced vasodepressor responses produced during an i.v. continuous infusion of methoxamine (20 μg/kg. min) (n = 5 for each group). No significant effects were produced after administration of compounds (P > 0.05). (PDF 881 kb) [file 10194_2018_869_MOESM1_ESM.pdf]

Additional file 1

(1 figure)

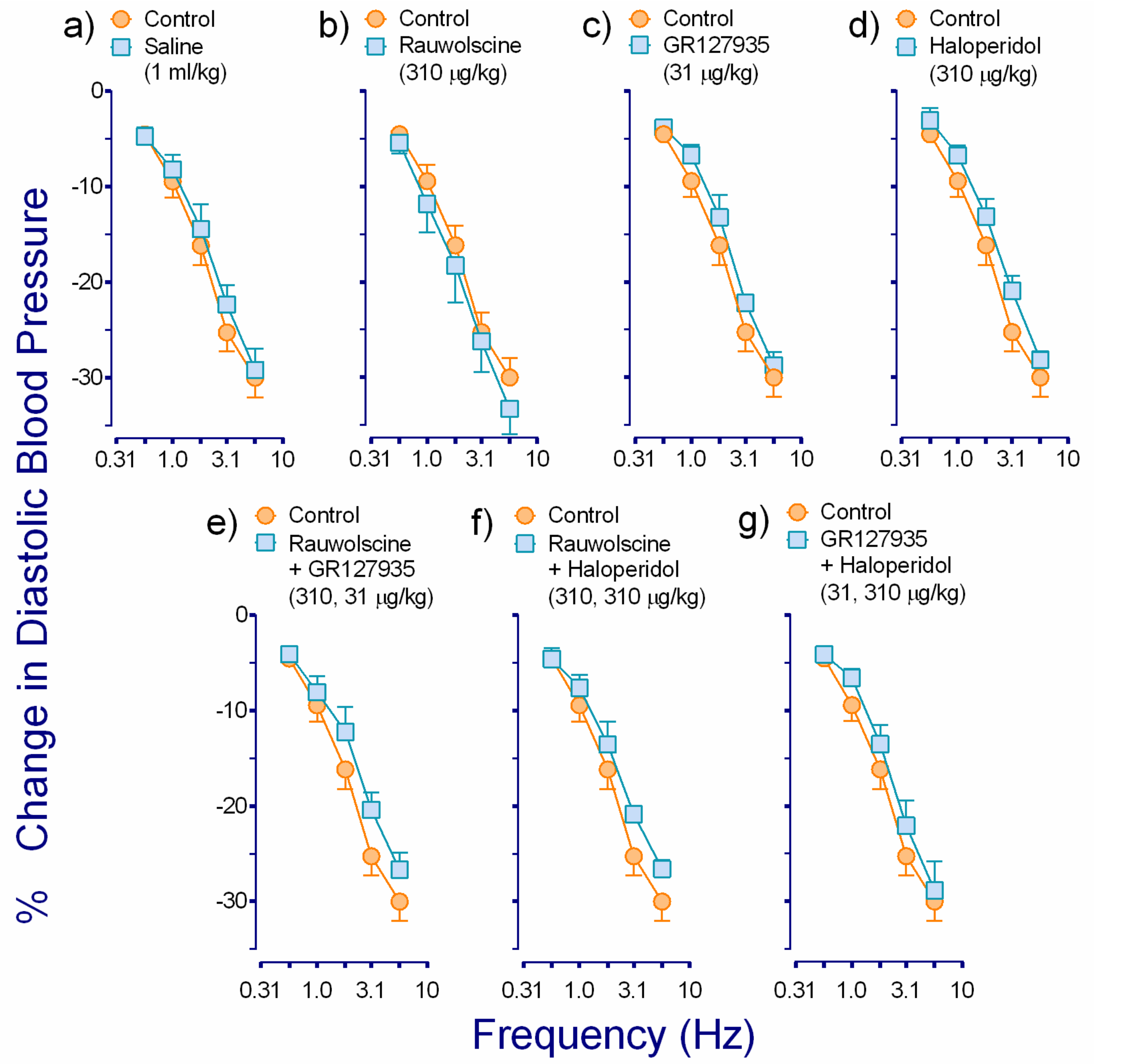

Figure S1. Effect *per se* of i.v. bolus injections of: (a) saline (1 ml/kg); (b) rauwolscine (310 μg/kg); (c) GR127935 (31 μg/kg); or (d) haloperidol (310 μg/kg) given separately; as well as the combinations (e) rauwolscine plus GR127935 (310 and 31 μg/kg, respectively); (f) rauwolscine plus haloperidol (310 μg/kg each); or (g) GR127935 plus haloperidol (31 and 310 μg/kg, respectively) on the electrically-induced vasodepressor responses produced during an i.v. continuous infusion of methoxamine (20 μg/kg. min) (n=5 for each group). No significant effects were produced after administration of compounds (P>0.05).
